# Supplementary figures and images for: Long non-coding RNA KIKAT/LINC01061 as a novel epigenetic regulator that relocates KDM4A on chromatin and modulates viral reactivation
Source: PLoS Pathog. 2021 Jun 10;17(6):e1009670. doi: 10.1371/journal.ppat.1009670 (PMC8219169; doi:10.1371/journal.ppat.1009670)

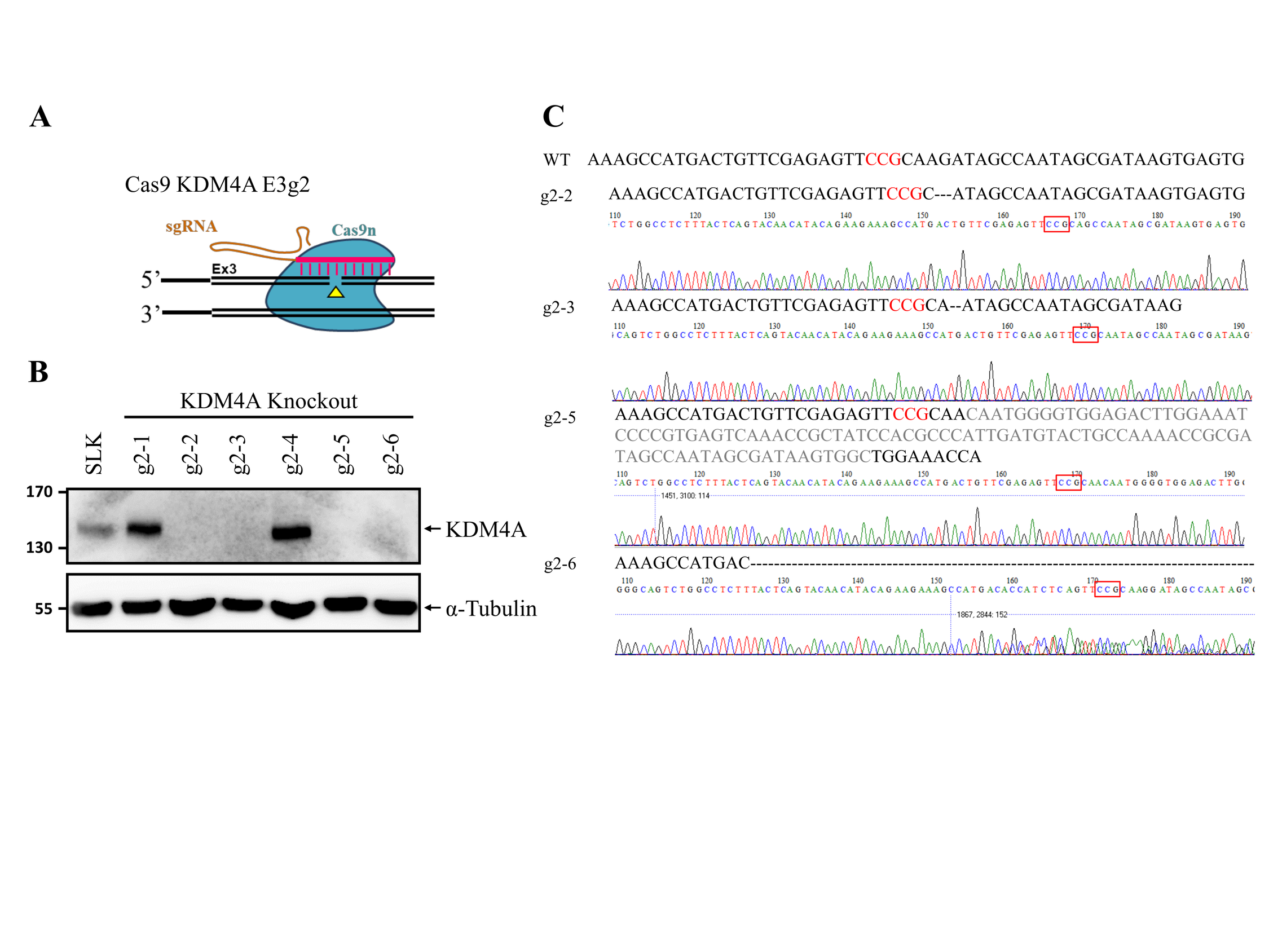

Supplement: S1 Fig — Generation of SLK KDM4A knockout cell line by CRISPR/Cas9 system (A) Schematic illustrating DNA breaks using Cas9 D10A nickases (Cas9n). The sequence of guide RNA targeting exon 3 of KDM4A (Cas9 KDM4A E3g2) was showed as follows: 5’-TAT CGC TAT TGG CTA TCT TGC GG-3’. (B) Immunoblotting of KDM4A in different KDM4A KO clones. (C) The knockout clones (g2-2, g2-3, g2-5, and g2-6) were confirmed by sequencing. Deleted sequences are indicted by dotted lines (Upper panel). The protospacer-adjacent motif (PAM) sequence is labeled in red rectangle. Chromatograms from Sanger sequencing of PCR amplicons over exon 3 of KDM4A are shown (Lower panel). (TIF) [file ppat.1009670.s001.tif]

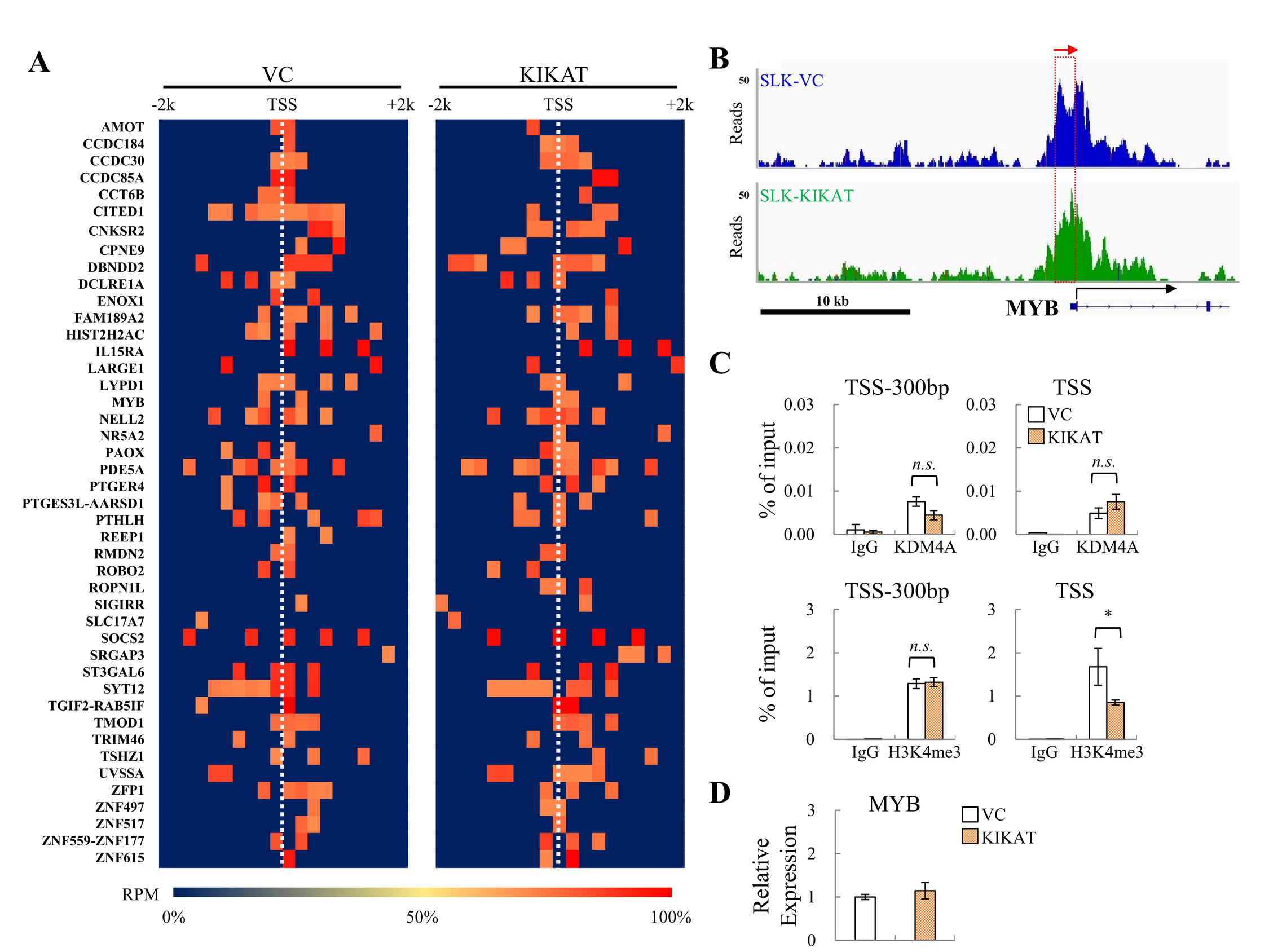

Supplement: S2 Fig — (A) Heatmap of ChIP-seq read density of KDM4A peaks in promoter region (TSS ± 2,000 bp) of KIKAT/LINC01061-regulated KDM4A-targeted genes. (B) Histograms of ChIP-seq profiles for KDM4A binding at MYB loci in SLK-VC and SLK-KIKAT/LINC01061 cells. (C) ChIP-qPCR assay revealed the binding of KDM4A (upper panel) and the modification of H3K4me3 (lower panel) to the promoter of MYB in SLK-VC and SLK-KIKAT/LINC01061 cells. (D) RT-qPCR analysis of MYB expression in SLK-KIKAT/LINC01061 cell lines. (TIF) [file ppat.1009670.s002.tif]

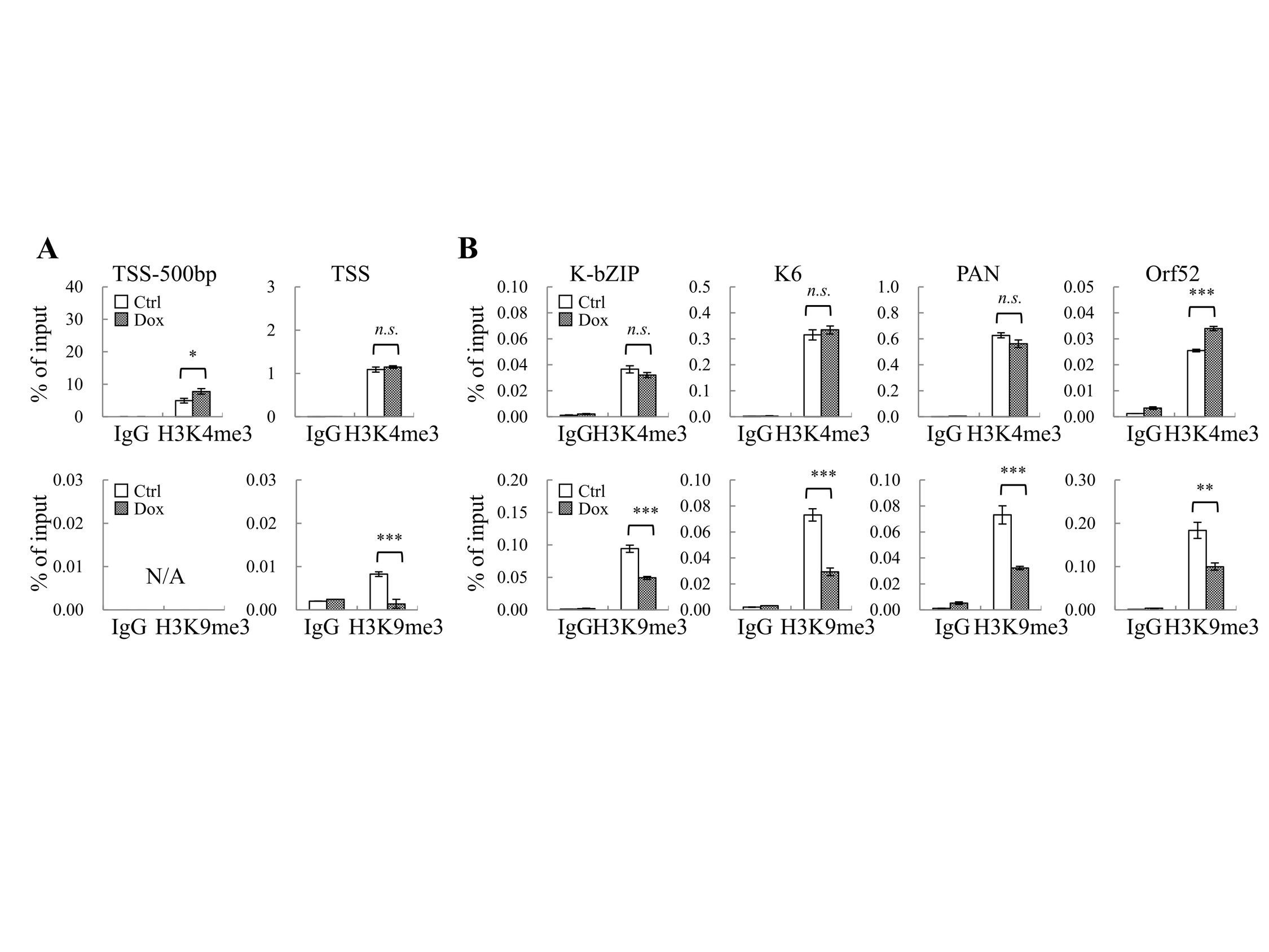

Supplement: S3 Fig — Histone marks alteration during KSHV reactivation (A) ChIP-qPCR assay revealed the modification of H3K4me3 (upper panel) and H3K9me3 (bottom panel) to the promoter of AMOT in iSLK-BAC16 cells treated with or without Dox for 72 hours. (B) ChIP-qPCR assay revealed the enrichment of H3K4me3 (upper panel) and H3K9me3 (lower panel) to the promoter of viral genes in iSLK-BAC16 cells before and after Dox induced KSHV reactivation for 72 hours. (TIF) [file ppat.1009670.s003.tif]

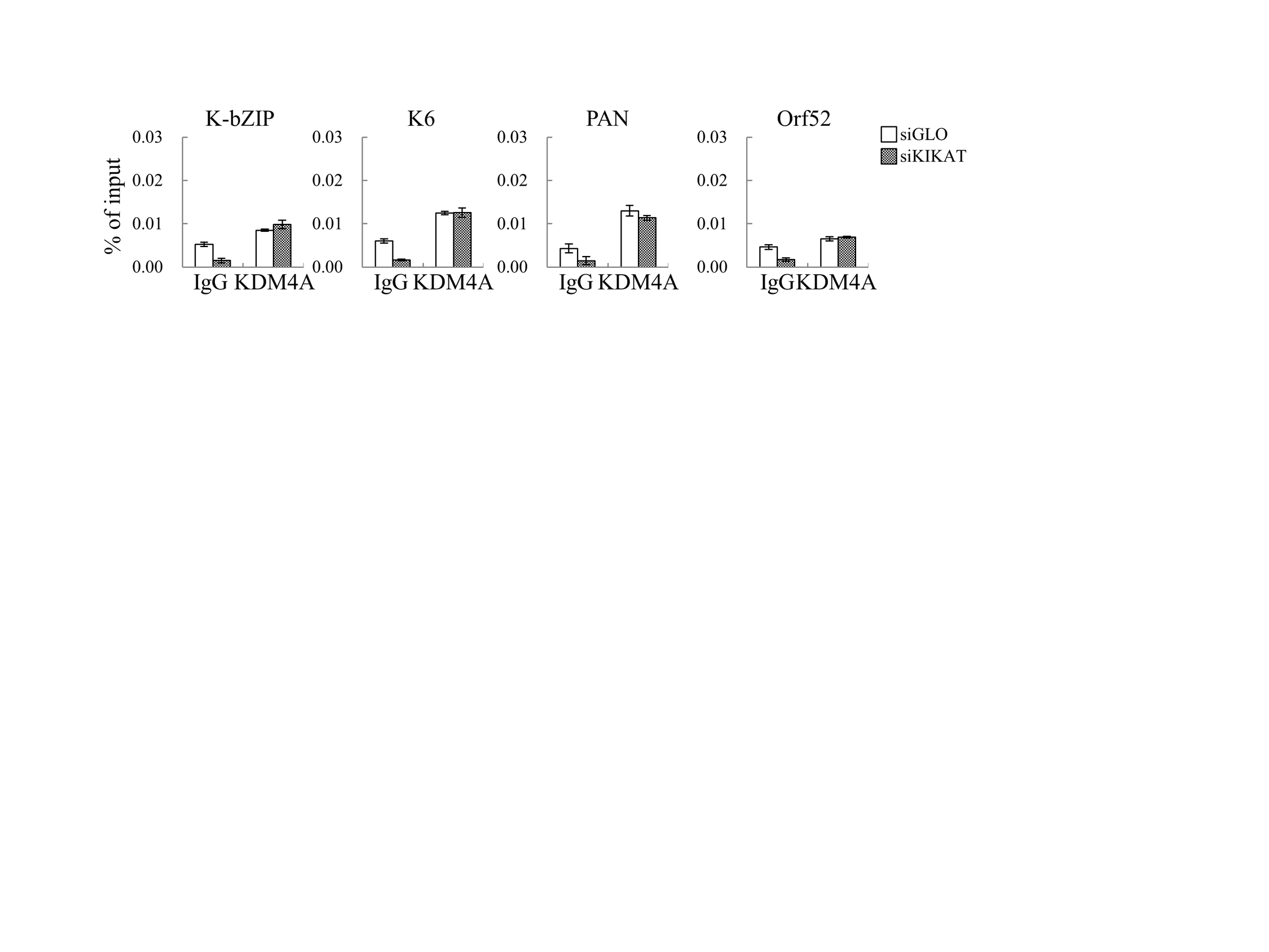

Supplement: S4 Fig — ChIP-qPCR assay revealed no changes in the enrichment of KDM4A on the promoter of select viral genes in TREx-F3H3-K-Rta BCBL-1 cells during latency before and after knockdown of KIKAT/LINC01061. (TIF) [file ppat.1009670.s004.tif]

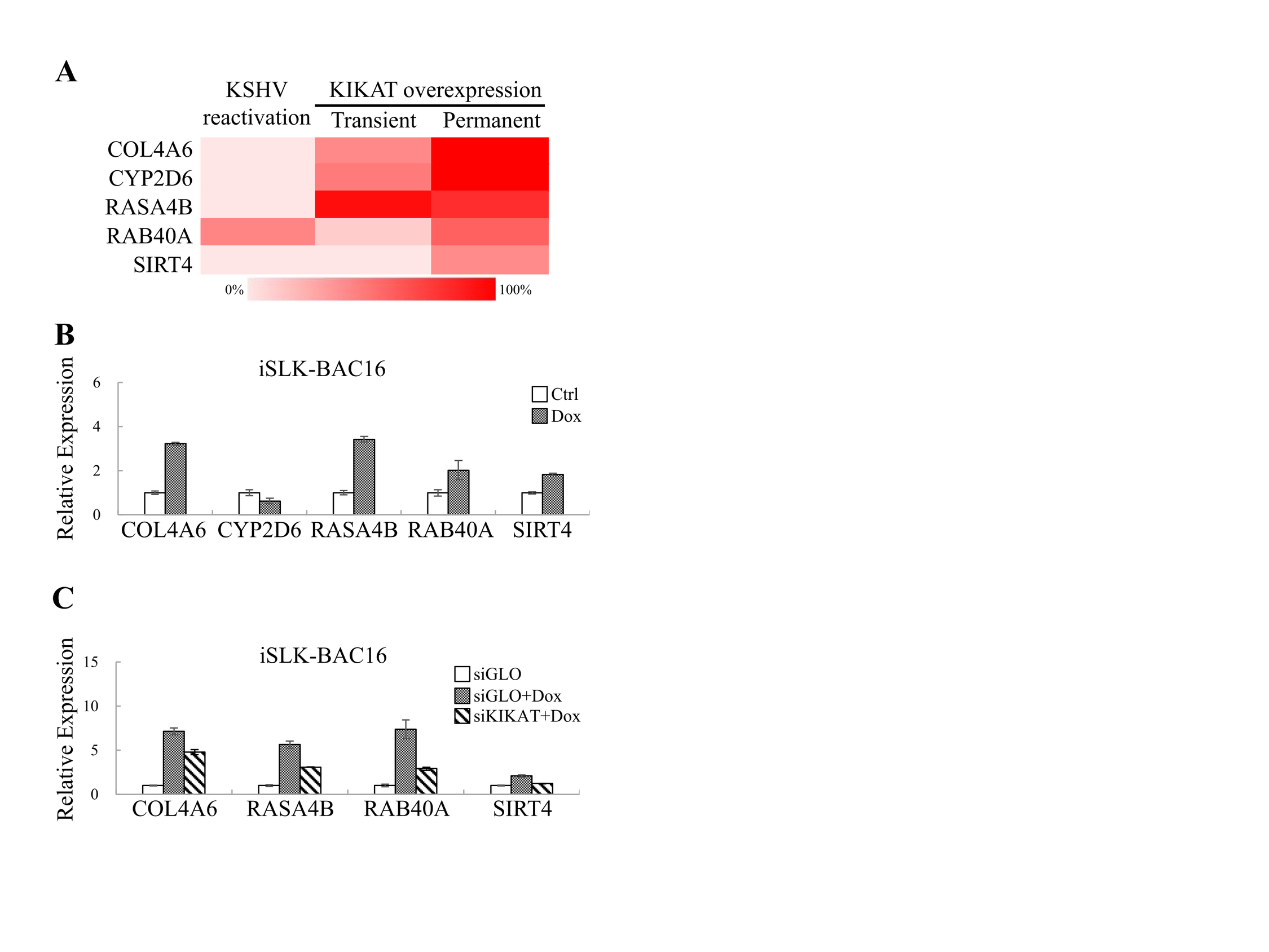

Supplement: S5 Fig — (A) Expression heatmap of top 5 up-regulated genes of RNA-seq data from KSHV reactivated iSLK-BAC16 cells and from transient and stable KAKIT/LINC01061 overexpression SLK cells. (B) RT-qPCR analysis of the expression of top 5 up-regulated genes identified in (A) in iSLK-BAC16 cells. (C) iSLK-BAC16 cells were transfected with siKIKAT/LINC01061 or with control siGLO. After 6 hours, cells were re-seeded in 6-well plate and treated with 1 μg/ml Dox for another 48 hours. RT-qPCR analysis of the 4 up-regulated genes identified in (B). (TIF) [file ppat.1009670.s005.tif]

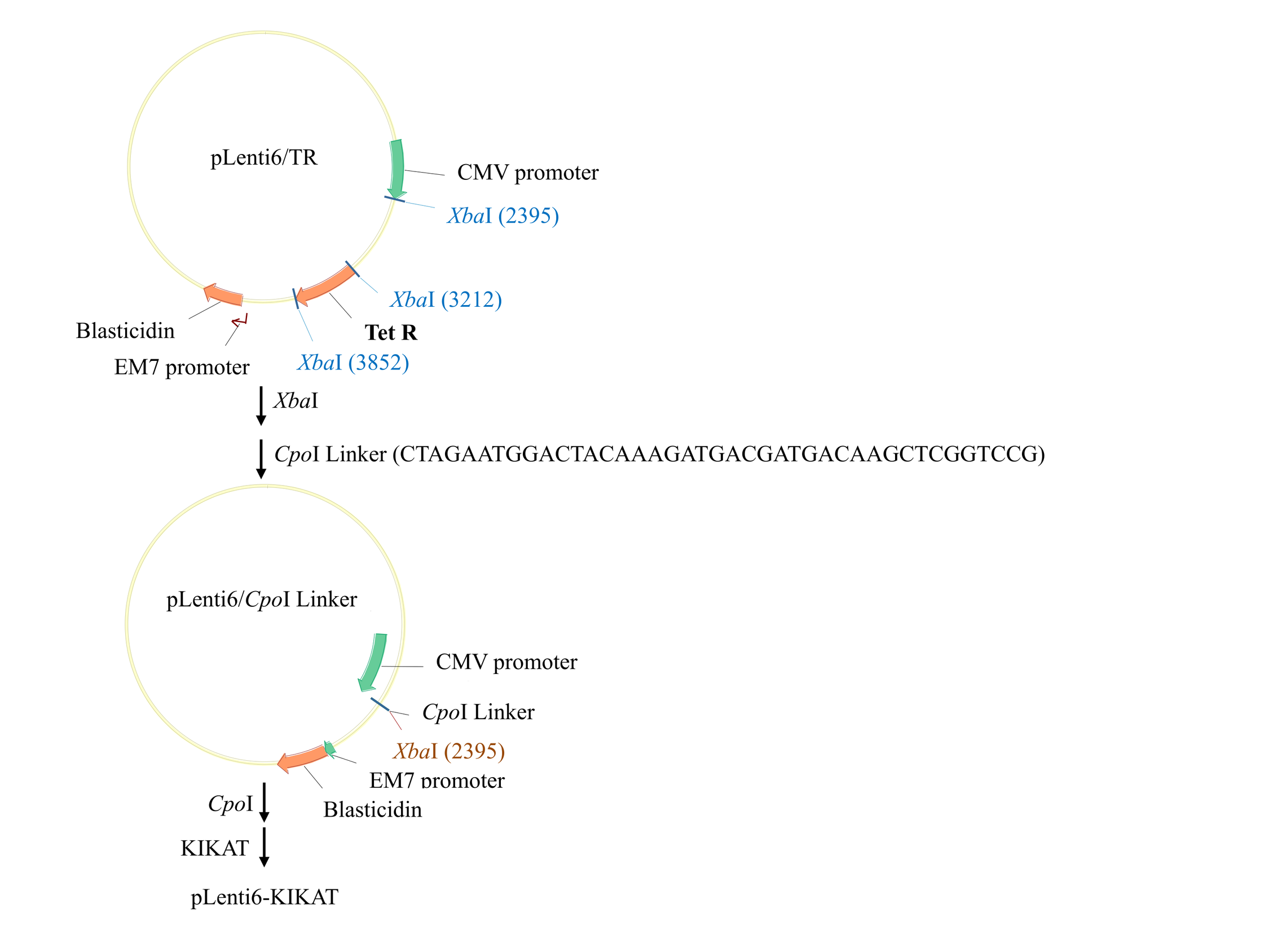

Supplement: S6 Fig — The pLenti6/TR lentiviral vector (Invitrogen, V48020) purchased from ThermoFisher was digested with XbaI to remove the TR, followed by insert a linker containing restriction site for CpoI. The KIKAT/LINC01061 cDNA digested by CpoI was cloned into the pLenti6-CpoI plasmid digested with the same enzyme. (TIF) [file ppat.1009670.s006.tif]
