# Supplementary material for: Long non-coding RNA KIKAT/LINC01061 as a novel epigenetic regulator that relocates KDM4A on chromatin and modulates viral reactivation
Source: PLoS Pathog. 2021 Jun 10;17(6):e1009670. doi: 10.1371/journal.ppat.1009670 (PMC8219169; doi:10.1371/journal.ppat.1009670)
Supplement: Attachment — Submitted filename: Revision_Letter_20210116_Final.docx [file ppat.1009670.s013.docx]

**PPATHOGENS-D-20-02267**

**Point by point response to reviewers**

We appreciate the reviewer’s comments, which have indeed significantly improved our manuscript. Following the journal’s review policy stating 「if more than 3 experiments are necessary to validate the study conclusions, then you are encouraged to recommend "Reject"」, our previous version of the manuscript was rejected. However, since laboratory work in Taiwan has not been affected by the pandemic issue of COVID-19, we were confident that the experiments suggested by the reviewers could be accomplished within 60 days. We asked the editors if a resubmission would be allowed and we were granted this opportunity. We performed the experiments requested and respond to each point from the reviewer’s suggestions as described below.

**Part II: Major Issues: Key Experiments Required for Acceptance**

**Reviewer #1:**

*1) In Fig. 1, the authors used the DOX-induced iSLK-BAC16 cells system to compare the transciptome levels of the KSHV latent and lytic cycle, and found the some lncRNA including LINC01061 are highly enhanced in lytic cycle. However, the iSLK is epithelial cell, and KSHV mainly infect endothelial cells or B cells to cause disease, whether this observation is associated with KSHV-associated cancers in physiological condition. They need to be confirmed in the endothelial and B cells with KSHV infection and lytic induction systems. Similar issues in Fig. 3.*

The comments are well taken. Following the reviewer’s suggestion, the expression of the 6 lncRNAs was also examined in (i) endothelial HMEC1 cells transduced with rKSHV.219 (Fig 1E) and (ii) KSHV(+) BCBL-1 cell line, TREx-F3H3-K-Rta BCBL-1 (Fig 1F), following K-Rta induction of KSHV reactivation. For the functional assays in Fig. 3, since we cannot successfully knockdown LINC01061 in BCBL-1 cells, the role of LINC01061 in mediating in KSHV reactivation was only analyzed in iSLK-BAC16. Your understanding is highly appreciated.

*2) In Fig.6 and Fig.7, to confirm whether the interaction of LINC01061 and KDM4A and their effect on cell migration is commonly occurred in cancers, the authors need to verify in couple of other type of cell lines, not just iSLK cell line.*

The reviewer’s comment is well taken. Following the reviewer’s suggestion, the interaction of LINC01061 and KDM4A was further confirmed in TREx-F3H3-K-Rta BCBL-1 cells (Figure 2D and 2E). The effect of LINC01061 on cell migration was further confirmed in HMEC1 & HUVEC (Figs. 7D and 7E).

*3) In Fig.7G, the schematic did not fully present the discovery of current data and absence of physiological relevance.*

Following the reviewer’s suggestion, we have removed Figure 7G from the manuscript.

**Reviewer #2:**

*(1) The title seems a little awkward and should be rephrased. Authors may consider “LicRNA LINC01061 is a epigenetic regulator and relocates KDM4A on chromatin”.*

The comment is well taken. We have modified our manuscript title accordingly.

*(2) Fig. 1. Through an approach of combination of RNA-seq and siRNA-mediated knockdown of gene expression, a group of genes were identified to be involved in KSHV reactivation.  This screening did not include any strategy to identify novel epigenetic regulatory lncRNA and should not claim “identification of novel epigenetic-regulatory incRNA … …” in the title of the section.*

This is a very good point. We have removed this description in the title of first section

*(3) Fig. 2. Why is KDM4A the only epigenetic enzyme among so many to be chosen for testing its interaction with these 6 lncRNAs?*

In 2015 and 2017, we published two KDM4A (JMJD2A)-related works in PLOS pathogens. In summary, we found that KDM4A is essential for KSHV reactivation. In continuing our research interest, we first chose KDM4A for testing its interaction with the 6 KSHV reactivation-related lncRNAs. For clarification, we have now included this statement in the beginning of the 2^nd^ section of results.

*(4) Fig. 2C. Linc01061 and Linc01341 can be detected in pretty high level in the precipitates by IgG. This suggests that the experiment needs to be repeated with a higher stringency wash.*

This is a very important point that needs to be taken into consideration. Following the reviewer’s comment, we repeated this experiment using a more vigorous washing process. It indeed improved the signal-to-noise ratio of LINC01061 (new data in Fig. 2C). New washing process was also used for New data of Fig. 2E.

*(5) Throughout the manuscript, there are a lot of overstatements and speculations that have no evident to support. For example, based on that Linc01601 was shown to down regulate ~500 genes (Fig. 4), authors stated that LINC01061 may reduce tumor suppressor genes in skin cancer and KS lesions. These speculations should be omitted, at least in the Result section.*

The comments are well taken. We have removed “the sentence” and other speculation from the result section.

*(6) The Linc01061-guided KDM4A shifting on chromatin is the most exciting discovery in this study. In the Table II, in some genes, KDM4A shifts from TSS to the upstream (such as AMOT), in others, KDM4A shifts from upstream to TSS (such as MYB). It is worthy to pick up a few representatives to explore the biological significance of the KDM4A relocation. If the shift in AMOT leads to activation of the gene, what does the shift in the MYB promoter cause?*

We appreciate the reviewer’s recognition of our exciting discovery. The comment is a very good point which we missed in our previous version. The KDM4A binding and histone marks of H3K4me3 on MYB promoter was analyzed by ChIP-qPCR. The expression of MYB upon LINC01061 overexpression was also studied by RT-qPCR (new data in Fig. S3). A change in MYB expression did not accompany KDM4A re-localization. We have added text (page 9, lines 15-22) to include these results and we state that LINC01061-mediated relocation of KDM4A to TSS at the MYB locus resulted in unchanged gene expression (page 9 lines 29-31)

**Reviewer #3:**

*iSLK-BAC16 reactivation for 24 hours with Dox alone is usually quite insufficient. What was the efficiency of KSHV reactivation? The subsequent RNA-seq analysis was done at 24 hpi. What stage of the lytic cycle does 24 hpi represent?*

Our view is similar to the reviewer that iSLK-BAC16 reactivation for 24 hours with Dox is at the beginning stage of KSHV reactivation before robust viral DNA replication. Following the reviewer’s suggestion, we have now included KSHV viral titer, as well as RT-qPCR and Western blot of K-bZIP, orf45 (IE gene), orf57 (E gene) and K8.1 (Late gene) in Figure 3.

*Statistics is missing at all qPCR graphs.*

This is a very good point which we missed in our previous version. We have added it according to the reviewer’s suggestions.

*Fig 1D What lncRNA was knockdown by siRNA?*

It is LINC01061 knockdown result and we have added it in Figure 1C (GFP/RFP images).

*RFP measurement is not that reliable for detecting changes in lytic reactivation since PAN promoter driving the expression of RFP can also be regulated independently of RTA. Thus, siRNA knockdown of some lncRNAs can give false positive RFP signal.*

Yes, we agree with the reviewer that siRNA knockdown of some lncRNAs can give a false positive RFP signal. However, this is an easy and cost-effective model for a middle to large scale screening. The screening data requires further confirmation. Here, we validate the essential role of LINC01061 in KSHV reactivation by knockdown and overexpression LINC01061 and detecting KSHV viral titers in Figure 3F and 3G, respectively.

*Fig 2: While KDM4A seems to be more abundant in SLK but the endothelial cells also express KDM4A well. It would be important to test/confirm if LINC01061 also interacts with KDM4A in biologically more relevant cell types and not only in uninfected but also in infected cells.*

*Besides SLK and endothelial cells, how about BCBL1 or other KSHV+ PEL cells during latency and reactivation?*

*Does KDM4A directly interact with LINC01061?*

The reviewer’s comments regarding additional cell types are well taken. Following the reviewer’s suggestion, we confirmed the LINC01061 and KDM4A interaction in KSHV(+) TREx-F3H3-K-Rta BCBL-1 cells (New data in Fig. 2D and 2E).

We also examined the expression levels of LINC01061 as well as the other 5 KSHV reactivation related lncRNAs in latent and lytic reactivated TREx-F3H3-K-Rta BCBL-1 cells (New data in Fig. 1F).

In our hands, we were unable to *in vitro* transcribe full length LINC01061. We therefore analyzed the interaction of KDM4A and LINC01061 by RIP. The RIP result cannot tell us if the interaction is direct or not. Your understanding is highly appreciated.

*Fig 3: It is unclear how Dox-inducible LIN01061 was made using pLenti6 vector because pLenti6 is just for constitutive expression of Tet repressor. Since iSLK cell line already expresses Tet repressor, pLenti6 is not required.*

*Also, the experiment in Fig 3E does not answer the question if LINC01061 alone induces viral reactivation because Dox treatment of iSLK-BAC16-LINC01061 cells will induce not only the lncRNA but also Dox-inducible RTA transgene, which will reactivate the virus.*

*It is surprising that Dox alone can produce so large amount of virus from iSLK-BAC16 cell at 72 hpi despite that the induction of IE/E gene K8 was only a few folds.* Adding the analysis of viral protein expression (some E and L proteins) by western blots would be helpful and their data would be more convincing.

We apologize for the misleading details in the clone construction. To constitutively overexpress LINC01061 by lentivirus, we excised the TR from pLenti6-TR vector using XbaI and the pLenti6 vector without TR was generated by self-ligation. After insertion a linker containing CpoI site into pLenti6 vector, LINC01061 was inserted into pLenti6 behind the CMV promoter. The cloning procedure is included in Supplementary Figure S4.

This is a very good point regarding whether LINC01061 can induce viral reactivation. Following the reviewer’s suggestion, the viral titer was quantified in iSLK-BAC16 following transient transduced lentiviral expressing LINC01061. LINC01061 alone does not induce KSHV reactivation (Fig. 3H). (New data in Fig 3H).

We have added text describing this experiment (page 7, lines 5-13).

Following the reviewer’s comment concerning the degree of Dox-induced viral reactivation, we have performed the immunoblotting of several viral proteins and the result showed significantly increased of K-bZIP (IE), orf45 (IE), orf57(E) and K8.1 (L) at 72 hpi (New data in Fig 3B and 3C).

*Fig 4: It is not explained why both lenti-LINC01061-transduced SLK cells and SLK-LINC01061 stable cell line were used for RNA-seq. Their gene expression profiling was performed separately and they were not compared to each other. Are there any common genes that were regulated by LINC01061 overexpression regardless of which cell line was used? Heat map visualization of the results would be helpful and also RT-qPCR confirmation of some of their top hits.*

*It is unclear how this dataset is connected to KSHV-infected cells and to their results presented in Figs 1-3. How LINC01061-regulated genes are expressed in iSLK-BAC16 cells or in other KSHV+ cell during latency and lytic reactivation? Does overexpression or the siRNA knockdown of LINC01061 affect the expression of genes in KSHV+ cells that were identified as LINC01061-regulated genes in the SLK-LINC01061 cell lines?*

Following the reviewer’s comment, we have added “both transient and constitutive LINC01061 overexpressed SLK cells were comprehensively searched” in the 1^st^ sentence of results section 3. Presentation of transient and constitutive LINC01061 overexpression up-regulated genes is visualized by heat map (New data in Fig. 4D). RT-qPCR of genes up-regulated by transient and constitutive overexpression of LINC01061 was performed (New data in Fig. 4E).

The reviewer is correct regarding the datasets. There is no connection between Fig 1-3 and the following figures. Since the gene expression alterations are complicated in the context of viral infection, we therefore elucidated the cellular function of LINC01061 in a viral free setting. Your understanding is highly appreciated.

*Fig 5 and 6: LINC01061 overexpression did not show a major effect on KSHV reactivation. Also, LINC01061 expression can already be high enough in cells so its overexpression may not affect KDM4A. The siRNA knockdown of LINC01061 would be better approach to test the effect of LINC01061 on KDM4A localization and function of KDM4A.*

*The 500-bp shift of KDM4A at AMOT promoter cannot be seen in Fig 6A as it is stated in the Result section. Could they use some graph, heatmap or other tools to visualize the shift of KDM4A localization at the 44 genes they identified?*

*ChIP-qPCR of KDM4A shows only reduction of KDM4A in SLK-LINC01061 cells but if there is a shift in KDM4A binding, it should also be shown on the ChIP graph. KDM4A enrichment should be increased in another location?*

*It is not obvious from the ChIP data how they concluded that LINC01061 and KDM4A protect H3K4me3 at AMOT from H3K9me3 invasion. Overexpression of LINC01061 has no effect on H3K9me3.*

*Here, it would be useful to include LINC01061 siRNA knockdown followed by ChIP for KDM4A, H3K9me3 and H3K4me3 to test if the absence of LINC01061 affects the distribution and levels of H3K9me3 and H3K4me3 at AMOT.*

Following the reviewer’s comment, we performed KDM4A ChIP assay in control and LINC01061 knockdown SLK cells. siRNA knockdown of LINC01061 has little effect on KDM4A binding (New data in Fig. 6C). This result indicated that basal expression level of LINC01061 may not be involved in chromatin binding of KDM4A.

The reviewer’s comment is well taken. We have now added the enrichment of KDM4A (RPM from ChIP-seq) in relevant to TSS in the absence and presence of LINC01061 overexpression in Fig. S3A.

The comment is well taken. Following the reviewer’s comment, we performed ChIP-qPCR of KDM4A binding and an increase of KDM4A on TSS -500bp of AMOT upon LINC01061 overexpression was observed (New data in Fig. 6B, left panel).

Yes, overexpression of LINC01061 resulted in reduction of KDM4A on TSS of AMOT without increase of H3K9me3 mark (Fig 6B). In contrast knockdown of KDM4A resulted in increase in H3K9me3 mark in AMOT promoter (Fig 6H). We therefore speculate that LINC01061 mediated KDM4A relocation protects H3K4me3 at AMOT from H3K9me3 invasion.

Following the reviewer’s comment, we performed KDM4A ChIP assay in control and LINC01061 knockdown SLK cells. Since knockdown of LINC01061 has little effect on KDM4A binding (New data in Fig. 6C), therefore we did not further perform ChIP assays for histone markers. Your understanding is highly appreciated.

*Fig 7: These results are interesting but they are not connected to KSHV given that LINC01061 was identified through its effect on KSHV. Is AMOT also upregulated in KSHV-infected cells during lytic reactivation in LINC01061-dependent manner? Does LINC01061 and AMOT regulate migration or spindle formation of KSHV-infected cells?*

We appreciate the reviewer’s recognition of our discovery as interesting. The reviewer’s comment is well taken. LINC01061 is also essential for the migration of KSHV-infected cells (New data in Fig. 7C). However, as mentioned above, the gene expression in the viral context is complicated. The RT-qPCR result showed that AMOT is not up-regulated in KSHV-infected cells during lytic reactivation. Therefore, LINC01061 mediated cell migration may be independent of AMOT in the context of viral infection.

Part III – Minor Issues: Editorial and Data Presentation Modifications

***Reviewer #1:*** *The authors need to describe what the potential difference of interaction of LINC01061 and KDM4A in oncovirus or non-oncovirus-mediated tumorigenesis in the discussion section.*

This is a very good comment. We have included this point in the discussion section (in the end of the 3^rd^ paragraph of the discussion section; page 12, lines 10-31)

**Reviewer #2**: (No Response)

***Reviewer #3****: It would be helpful to the reviewers if they had included line numbers and page numbers in the manuscript.*

*In the Abstract, SLK is described as cells revived form KS. However, it has been proven that SLK cells are not originated from KS [Sturzl et al (Int J Cancer, 2013, 132(8):1954-8].*

*It would be interesting to know if RTA expression alone (using Dox-induced iSLK cells) can also induce the lncRNAs seen in iSLK-BAC16 cells. It would strengthen their finding that a viral factor during viral reactivation induces the upregulation of host lncRNAs.*

We apologize for the inconvenience. Following the reviewer’s suggestion, we have included line numbers and page numbers in the manuscript

The reviewer’s comment is well taken. We have removed the sentence from the Abstract.

It is a very insightful comment. As reviewer’s expectation, RT-qPCR and reporter assays confirmed that LINC01061 is induced by K-Rta (New data in Fig. S1).

*In Discussion: “…we identified 6 epigenetic-regulatory lncRNA essential for opening the viral DNA (Table S1 and Fig. 1D) using entry into the viral lytic cycle as readout.” – Rephrase it because it is not clear what they mean by it.*

*“As an oncogenic virus-induced epigenetic-regulatory lncRNA essential for opening the viral DNA (Fig. 3), we suspect that induction of LINC01061 may be closely related to tumorigenesis.”- How does the role of a lncRNA in the regulation of viral chromatin links the lncRNA to oncogenesis? It is better to rephrase the sentence.*

*There are several typos throughout the paper.*

We have carefully edited the revised manuscript and hopefully rectified all the previous problems.
