# Supplementary material for: Long non-coding RNA KIKAT/LINC01061 as a novel epigenetic regulator that relocates KDM4A on chromatin and modulates viral reactivation
Source: PLoS Pathog. 2021 Jun 10;17(6):e1009670. doi: 10.1371/journal.ppat.1009670 (PMC8219169; doi:10.1371/journal.ppat.1009670)
Supplement: Attachment — Submitted filename: Revision_Letter_20210407.docx [file ppat.1009670.s014.docx]

**PPATHOGENS-D-21-00117R1**

**Point by point response to reviewers**

We appreciate the reviewer’s comments that have indeed significantly improved the focus and readability of our manuscript. Following the wonderful comments to study “*if the results from LINC01061 overexpression/knockdown experiments in KSHV-free cells are applicable to KSHV-infected cells.*”, we performed 6 additional experiments suggested by the reviewers. Accordingly, this manuscript has undergone a 2nd major revision and our response to each point from the reviewer’s suggestions are described below. We have attempted to address previous criticism regarding the use of both KSHV-free and -infected cells in this revision. Using each of the reviewer’s guidance and suggestions, we now feel that we have obtained the additional data needed that allows for us to combine both KSHV-free and -infected conditions within a single manuscript.

**Part II: Major Issues: Key Experiments Required for Acceptance**

**Reviewer #1:**

*(1) The title indicates that the non-coding RNA is important for viral reactivation. However, the whole article does not address if LINC01061 has any role in KSHV reactivation. Authors have done RNA-seq on LINC01061-over-expressed KSHV-SLK cells and ChIP-seq for KDM4A. Why don’t authors map the reads to the KSHV genome? The results will show the effects of the noncoding RNA on viral gene expression and if LINC01061 relocates KDM4A on the viral chromatin.*

We apologize for the misleading. Both RNA-seq (Fig. 3) and ChIP-seq (Fig. 4) was done in SLK cells without KSHV infection. We agree with the reviewer and have (i) added the up-regulation of KIKAT/LINC01061 by K-Rta (Fig. 8A to D) during KSHV reactivation (Fig. 7D) and (ii) showed that KIKAT/LINC01061 is essential for viral reactivation using knockdown and overexpression experiments, (Fig. 8E and 8F).

**Fig. 7D** Expression of KIKAT/LINC01061 in iSLK-BAC16 cells treated with 1 μg/ml Dox for 0, 24, 48 and 72 hours were determined by RT-qPCR.

**Fig. 8A to 8D** (A) Immunoblotting of K-Rta in iSLK cells treated with or without Dox. (B) RT-qPCR analysis of KIKAT/LINC01061 in iSLK cells treated as described in (A). (C) The reporter plasmid containing KIKAT/LINC01061 promoter was co-transfected with pcDNA3-Flag-K-Rta or empty vector into 293T cells. Cells were collected for immunoblotting with anti-HA and anti-α-Tubulin antibodies. (D) Reporter assays were performed in 293T cells as described in (C).

**Fig. 8E** iSLK-BAC16 cells were transfected with siKIKAT/LINC01061 or with control siGLO. After 6 hours, cells were re-seeded in 6-well plates and treated with 1 μg/ml Dox for another 48 hours. Relative expression of KIKAT/LINC01061 (upper panel) and KSHV virion-associated DNA (lower panel) was determined by RT-qPCR and TaqMan qPCR, respectively. **Fig. 8F** iSLK-BAC16 cells were transduced with lentivirus expressing full-length KIKAT/LINC01061 or with no cDNA (Mock). After 16 hours, cells were re-seeded in 6-well plates and treated with 1 μg/ml Dox for another 72 hours. Relative expression of KIKAT/LINC01061 (upper panel) and KSHV virion-associated DNA (lower panel) was determined as described in (E).

To show if KIKAT/LINC01061 relocates KDM4A on the viral chromatin, we performed ChIP-qPCR in control and Dox-treated iSLK-BAC16 cells with or without siKIKAT/LINC01061 transfection (Fig. 9B, Fig. 9C and Fig. S3B).

**Fig. 9B** iSLK-BAC16 cells transfected with siKIKAT/LINC01061 or with control siGLO were subjected to ChIP assay and the binding of KDM4A to the promoter of AMOT were analyzed by qPCR.

**Fig. 9C** The enrichment of KDM4A on the promoter of viral genes were analyzed by qPCR.

**Fig. S3B** ChIP-qPCR assay revealed the enrichment of H3K4me3 (upper panel) and H3K9me3 (lower panel) to the promoter of viral genes in iSLK-BAC16 cells before and after Dox induced KDSHV reactivation for 72 hours

*(2) Fig. 6A and Fig. S3B, the KDM4A relocation on AMOT and MYB genes needs to be indicated by arrows.*

Following the reviewer’s suggestion, we have added arrows to show the relocation of KDM4A.

*(3) The manuscript can be written more concisely, which will improve the readability of the manuscript. The title needs to be revised.*

The reviewer’s comment is well taken. We have revised the title and re-written the manuscript to improve the readability. The modifications are highlighted in the revised manuscript with tracking.

**Reviewer #2:**

*(1) Based on their current findings it is unclear how much of the results from LINC01061 overexpression/knockdown experiments in KSHV-free cells are applicable to KSHV-infected cells. The question remains how many of the genes, which are regulated by LINC01061, are also controlled by LINC01061 in KSHV-infected cells? AMOT is regulated by KDM4A/LINC01061 in SLK cells and affects angiogenesis but not in KSHV-infected cells? RNA-seq analysis of lytic reactivated KSHV-infected cells in which LINC01061 expression is depleted by shRNA could reveal how LINC01061 contributes to KSHV life cycle and the expression of tumorigenesis pathway genes in KSHV+ cells.*

The reviewer’s comment is well taken. We have compared the differentially expressed genes (DEGs) in KIKAT/LINC01061 overexpression experiments (Fig. 3) with DEGs in KSHV reactivated iSLK-BAC16 cells (Fig. 1). 32% of the up-regulated genes in both transient and constitutive KIKAT/LINC01061 overexpression SLK cells were also up-regulated during KSHV reactivation. The top 5 genes commonly upregulated were analyzed in iSLK-BAC16 cells by RT-qPCR. Four of top 5 KIKAT/LINC01061 activated genes were also up-regulated by KSHV reactivation and the up-regulation of those genes were diminished to varying extents by knockdown of KIKAT/LINC01061 (Fig. S4).

**Fig. S4** Identification and verification of KIKAT/LINC01061-activated genes in iSLK-BAC16. **(A)** Expression heatmap of top 5 up-regulated genes of RNA-seq data from KSHV reactivated iSLK-BAC16 cells and from transient and stable KAKIT/LINC01061 overexpression SLK cells. **(B)** RT-qPCR analysis of the expression of top 5 up-regulated genes identified in (A) in iSLK-BAC16 cells. (**C**) iSLK-BAC16 cells were transfected with siKIKAT/LINC01061 or with control siGLO. After 6 hours, cells were re-seeded in 6-well plate and treated with 1 μg/ml Dox for another 48 hours. RT-qPCR analysis of the 4 up-regulated genes identified in (B).

Following the reviewer’s suggestion, we assessed chromatin binding alterations of KDM4A in the presence or absence of KIKAT/LINC01061 during KSHV reactivation. KDM4A relocation was observed on AMOT promoter region (Fig 9B) after KSHV reactivation. We also performed transwell assays and the result showed that knockdown of KIKAT/LINC01061 significantly inhibited SLK migration induced by viral reactivation (Fig. 9D).

**Fig 8E** iSLK-BAC16 cells were transfected with siKIKAT/LINC01061 or with control siGLO. After 6 hours, cells were re-seeded in 6-well plates and treated with 1 μg/ml Dox for another 48 hours. Relative expression of KIKAT/LINC01061 (upper panel) and KSHV virion-associated DNA (lower panel) was determined by RT-qPCR and TaqMan qPCR, respectively.

**Fig 9B** iSLK-BAC16 cells transfected with siKIKAT/LINC01061 or with control siGLO were subjected to ChIP assay and the binding of KDM4A to the promoter of AMOT were analyzed by qPCR.

**Fig 9D** iSLK-BAC16 cells were transfected with control siGLO or siKIKAT/LINC01061. After 6 hours, cells were re-seeded in 6-well plate and treated with 1 μg/ml Dox for another 48 hours. *in vitro* transwell migration analysis was determined. Representative images (100x magnification) of transwell migration analysis (left panel). Scale bar: 100 μm. Quantification of cell migration (right panel).

Following the reviewer’s suggestion, we performed knockdown experiment to showed that KIKAT/LINC01061 is essential for viral reactivation (Fig. 8E).

*(2) Also, the group has published excellent studies on how KDM4A binds to the KSHV genome and affects lytic gene expression. Does LINC01061 also affect KDM4A and H3K4me3/H3K9me3 enrichment/ratio on the viral chromatin like on the host genome?*

The reviewer’s comment is well taken. Following the reviewer’s comment, we performed ChIP of KDM4A (Fig. 9C) and H3K4me3/H3K9me3 (Fig. S3B) in control and Dox-treated iSLK-BAC16 cells. qPCR was performed to detect KDM4A binding on viral promoters that we identified in our previous report.

**Fig. 9C** The enrichment of KDM4A on the promoter of viral genes were analyzed by qPCR.

**Fig. S3B** ChIP-qPCR assay revealed the enrichment of H3K4me3 (upper panel) and H3K9me3 (lower panel) to the promoter of viral genes in iSLK-BAC16 cells before and after Dox induced KSHV reactivation for 72 hours.

**Reviewer #3:**

*1) The title of manuscript is not appropriately to summarize and highlight what the current data has presented. Since the majority study was started by lncRNA screening of KSHV lytic reactivation, and then found that the lncRNA LINC01061 was induced by KSHV lytic reactivation for binding to KDM4A and relocation of KDM4A for increasing AMOT expression and migration in SLK cells without KSHV infection. Due to KSHV lytic replication will lead to host cell destroy, why KSHV needs to induce LINC01061 expression in lytic replication instead of latency for oncogenesis? To highlight the physiological relevance of viral pathogenesis or just KDM4A oncogenicity, it would better to focus the role in viral reactivation or host cell effect only, but not both.*

We agree with the reviewer and have removed “*is important for viral reactivation*” from the title.

*2) In Fig.2B and D, it needs to compare the difference of KDM4A and its ability bound to LINC01061 before and after KSHV lytic reactivation, to confirm the conclusion that LINC01061 is specifically induced by KSHV reactivation and bound to KDM4A.*

This is a very good comment. Following the reviewer’s suggestion, we performed RIP in control and Dox induced iSLK-BAC16 cells and showed an increased in the interaction between KIKAT/LINC01061 and KDM4A in KSHV reactivated cells (Fig. 9A).

**Figure 9** KIKAT/LINC01061 and KDM4A association in iSLK-BAC16 cells. (**A**) TCLs from control and Dox induced iSLK-BAC16 cells were used for RIP using KDM4A antibody or rabbit IgG as control. Copurified RNA from KDM4A and IgG IPs was assessed for KIKAT/LINC01061 enrichment by RT-qPCR.

*3) In Fig.6, it needs to include the data to prove that KDM4A is also relocated on host cell chromatin after KSHV reactivation, not just SLK-VC and SLK-LINC01061 cell lines.*

The reviewer’s comment is well taken. Following the reviewer’s comment, we performed KDM4A ChIP in control and Dox-treated iSLK-BAC16 cells with or without siKIKAT/LINC01061 transfection. The result showed that KDM4A is also relocated on AMOT promoter after KSHV reactivation and that was mediated by KIKAT/LINC01061 (Fig. 9B)

**Fig 9B** iSLK-BAC16 cells transfected with siKIKAT/LINC01061 or with control siGLO were subjected to ChIP assay and the binding of KDM4A to the promoter of AMOT were analyzed by qPCR.

*4) In Fig. 7B, the author presented that LINCO1061 plays a role in cell migration by using SLK cell line. What is the role for KSHV to enhance LINC01061 expression during lytic replication? also enhancing lytic host cell migration? need to be addressed.*

This is a very good comment. Following the reviewer’s comment, we performed a knockdown experiment and showed that KIKAT/LINC01061 is essential for KSHV reactivation cell migration (Fig. 9D).

**Fig 9D** iSLK-BAC16 cells were transfected with control siGLO or siKIKAT/LINC01061. After 6 hours, cells were re-seeded in 6-well plate and treated with 1 μg/ml Dox for another 48 hours. *in vitro* transwell migration analysis was determined.

Part III – Minor Issues: Editorial and Data Presentation Modifications

**Reviewer #1**: (No Response)

**Reviewer #2**:

*Fig 1E, F Some comment would be useful on why most of the lncRNAs that are induced in RTA-expressing iSLK-BAC16 and BCBL1 were not upregulated in HMEC1-KSHV.r219 cells reactivated by RTA. Add these comments after these result were described.*

This is a very good point. We have added these comments after the description of the results (Page 6, line 9-10).

*P6 line 36 KHSV-DNA, misspelling of KSHV*

We apologized for the misspelling and corrected it in the new version.

*Fig 3C ORF57 western blot did not seem to work. I suggest that it be repeated or it can also be deleted.*

The reviewer’s comment is well taken. We have now deleted the ORF57 blot in the results.

*P6-7 “To this end, we first detected the KSHV titer in iSLK-BAC16 cell supernatants after K-Rta induction for 24, 48 and 72 hours (Fig. 3E).” This requires some conclusion before talking about the follow-up experiment.*

This is a very good point. We have added a conclusion after the description of these results (Page 10, line 16).

*P7 line 6-7 “…we further generated an inducible LINC01061 expressing cell line using iSLK-BAC16 cells, iSLK-BAC16-LINC01061.” As far as I understood the lncRNA was overexpressed in iSLK-BAC16 using lentiviral transduction and lytic reactivation was triggered by Dox so the lncRNA was not inducible. If so, I suggest that the sentence be changed.*

Yes, KIKAT/LINC01061 was not inducible. We apologized for the misleading description and have corrected it according to the reviewer’s suggestion in the new version (Page 10, line 31-32).

*Fig 4D-E The gene PALM seems to be strongly upregulated by LINC01061 based on the heat map but its RT-qPCR test shows no PALM expression upon LINC01061.*

According to the RNA-seq data, we suspected that this might due to the expression level of PALM is low (RPKM ~0 in control and ~0.2 upon LINC01061 overexpression).

*P7 line 28 I believe Fig 4E wants to show the confirmation of host genes that were upregulated by LINC01061. If so, “…was first checked by…” it should read “…was confirmed by…”*

The comment is well taken. We have modified the manuscript according to the reviewer’s suggestion (Page 7, line 11-13).

*P8 “…was also used to assess the tumorigenesis of LINC01061-repressed genes” it should read “…was also used to assess the association of LINC01061-repressed genes with tumorigenesis”*

We agree with the reviewer and have modified the manuscript accordingly (Page 7, line 21-22).

**Reviewer #3**: (No Response)

We have carefully edited the revised manuscript and hopefully rectified all the previous problems.
