# Supplementary material for: Long non-coding RNA KIKAT/LINC01061 as a novel epigenetic regulator that relocates KDM4A on chromatin and modulates viral reactivation
Source: PLoS Pathog. 2021 Jun 10;17(6):e1009670. doi: 10.1371/journal.ppat.1009670 (PMC8219169; doi:10.1371/journal.ppat.1009670)
Supplement: Attachment — Submitted filename: Revision_Letter_20210524.docx [file ppat.1009670.s015.docx]

**PPATHOGENS-D-21-00117R2**

**Point by point response to reviewers**

We are happy to know that the editor felt our manuscript “is likely to be acceptable for publication”. We appreciate that the reviewers think we have addressed most of the concerns raised in the last submission. Following the minor comments from two reviewers, we performed one additional experiment and made several modifications in the figures and text as suggested. Accordingly, this manuscript has undergone a 3rd revision and our response to each point from the reviewer’s suggestions are described below.

**Part I: Summary**

**Reviewer #1:**

*In this revision, the authors have addressed most of the concerns raised in the last submission. The data support the conclusion that LncRNA KIKAT is an epigenetic regulator that modulates viral reactivation and oncogenic phenotypes through binding to KDM4A. The manuscript sounds well in science, but the manuscript can be further polished to make it more concise and improve its readability. Figures 7 and 8 can be combined as there are too little data in each figure.*

We thank the reviewer for considering that our manuscript sounds well in science and that we have addressed most of the concerns raised in the last submission. We agree with the reviewer and have combined Figures 7 and 8.

**Reviewer #2:**

*The manuscript is improved after revision and the authors have performed additional experiments to address previous reviewers' question. One of the major questions was how LINC01061 affects KDM4A on the KSHV genome. Figure 9 and Figure S3 provided some new information but it is confusing. It seems that the siRNAkd of LINC01061 increases KDM4A binding at PAN promoter while the binding of KDM4A does not change at other viral promoters upon lytic reactivation. However, H3K9me3 is reduced at PAN while KDM4A binding increases. This is counterintuitive. In JVI 2011 Chang et al paper on JMJD2A/KDM4A, the binding of KDM4A was studied only during latency and in BCBL1. In the current manuscript the authors show in Figure 2E that KDM4A interacts with LINC01061 in BCBL1 during latency. Can they use latent BCBL1 cells and siRNA for LINC01061 to show if LINC01061 affects KDM4A binding to viral promoters that were shown in the JVI 2011 paper Chang et al? If LINC01061 does not affect KDM4A binding on the viral genome, it would indicate that LINC01061 may regulate KDM4A on host but not on viral genome, which is okay. This needs to be addressed in the discussion, which was omitted in the revised manuscript*

We appreciate the reviewer for considering the additional experiments we performed addressing the previous questions. Following the reviewer’s suggestion, we performed KDM4A ChIP in KIKAT/LINC01061 knockdown BCBL1 cells. As the reviewer expected, KIKAT/LINC01061 does not affect KDM4A binding on the viral genome. Results supporting reviewer’s comment were obtained and have now been added as Figure S3. We have added some text describing this experiment in the corresponding Results section (page 11 lines 26-31). We have also incorporated this result in our concluding summary in the Discussion (page 16 lines 14-17).

Identification of KDM4A binding on latent KSHV genome following knockdown of KIKAT/LINC01061. ChIP-qPCR assay revealed the enrichment of KDM4A on the promoter of viral genes in BCBL-1 cells before and after knockdown of KIKAT/LINC01061.

**Reviewer #3:**

*The authors have addressed most of concerns raised by the reviewers in the third revised manuscript, and it is now suitable for publication in the current version.*

We thank the reviewer for considering that we have addressed most of the concerns raised in the 3^rd^ revision and it is suitable for publication.

**Part II: Major Issues: Key Experiments Required for Acceptance**

**Reviewer #1:** (No Response)

**Reviewer #2:** (No Response)

**Reviewer #3:** (No Response)

**Part III: Minor Issues: Editorial and Data Presentation Modification**

**Reviewer #1:** (No Response)

**Reviewer #2:** (No Response)

**Reviewer #3:** (No Response)
